# Combustible Cigarette Smoking, Electronic Cigarette Use, and Metabolic Syndrome in a Working Population

**DOI:** 10.3390/epidemiologia7040100

**Published:** 2026-07-13

**Authors:** Astrid Lorena Urbano Cano, Rosa Elvira Alvarez Rosero, Nelson Esteban Portuguez Jaramillo, Jose Luis Piñeros-Alvarez

**Affiliations:** 1 Grupo de Investigación en Salud Integral (GISI), Facultad de Salud, Universidad Santiago de Cali, Cali 760035, Colombia; nelson.portuguez00@usaca.edu.co; 2 Grupo de Investigación en Genética Humana Aplicada (GIGHA), Departamento de Ciencias Fisiológicas, Universidad del Cauca, Popayan 190003, Colombia; ralvarez@unicauca.edu.co; 3 Grupo de Investigación en Genética, Fisiología y Metabolismo (GEFIME), Facultad de Salud, Universidad Santiago de Cali, Cali 760035, Colombia; jose.pineros00@usc.edu.co

**Keywords:** metabolic syndrome, electronic cigarettes, workers

## Abstract

Background: Metabolic syndrome is a major public health concern because of its association with cardiovascular disease and type 2 diabetes. Tobacco exposure, including combustible cigarette smoking and electronic cigarette use, has increasingly been linked to cardiometabolic dysfunction; however, evidence in occupational populations remains limited. Objectives: This study evaluated the association of combustible cigarette smoking, electronic cigarette use, and dual use with metabolic syndrome in a working population from southwestern Colombia. Methods: We conducted a cross-sectional study of 822 workers from formal companies. Tobacco exposure was self-reported and categorized as exclusive electronic cigarette use, exclusive combustible cigarette smoking, dual use, or non-use. Sociodemographic, anthropometric, and metabolic variables were collected using standardized procedures. Logistic regression models were fitted to estimate crude and adjusted odds ratios with 95% confidence intervals. Results: Metabolic syndrome was identified in 39.8% of participants. It was more frequent in men, who also exhibited a greater accumulation of metabolic abnormalities. Hypertriglyceridemia and low high-density lipoprotein cholesterol were the components most strongly associated with metabolic syndrome. Compared with non-use, the odds of metabolic syndrome were higher across all tobacco-exposed groups, with odds ratios of 3.02 for exclusive electronic cigarette use, 7.29 for exclusive combustible cigarette smoking, and 4.75 for dual use. The strongest association was observed for exclusive combustible cigarette smoking. Conclusions: Metabolic syndrome was prevalent in this occupational population, and tobacco-related exposures were associated with a less favorable cardiometabolic profile. These findings support integrated workplace prevention strategies that combine smoking control with early detection of metabolic risk.

## 1. Introduction

Metabolic syndrome (MetS) is a cluster of cardiometabolic abnormalities that includes central obesity, elevated blood pressure, impaired glucose metabolism, hypertriglyceridemia, and low levels of high-density lipoprotein (HDL) cholesterol [1]. This condition is a clinical and epidemiological marker of increased risk, as it is associated with a doubling of the risk of cardiovascular disease (CVD) and up to a fivefold increase in the risk of developing type 2 diabetes mellitus (T2DM) [2].

In recent decades, the burden of MetS has increased significantly. A recent global analysis showed that, between 2000 and 2023, the global prevalence of MetS increased from 14.7% to 31.0% in women and from 9.0% to 25.7% in men, with marked heterogeneity according to sex, socioeconomic status, urbanization, and geographic region. By 2023, it was estimated that approximately 1.54 billion adults worldwide were living with MetS [3].

This increase likely stems from the interaction between population aging, urbanization, and lifestyle changes characterized by high-energy diets, reduced physical activity, increased sedentary behavior, and greater exposure to psychosocial stress [3,4]. In this context, MetS is a priority public health issue due to its close relationship with the epidemiological transition and the accumulation of cardiometabolic risk factors.

Among the modifiable behavioral factors associated with cardiometabolic risk, tobacco use is a significant factor. Accumulated evidence suggests that conventional cigarettes are associated with an increased risk of MetS and alterations in some of its components, possibly through mechanisms related to inflammation, oxidative stress, endothelial dysfunction, and alterations in lipid and carbohydrate metabolism [5,6].

More recently, e-cigarette use has emerged as a contemporary form of nicotine exposure, frequently perceived as less harmful than conventional cigarettes [7]. International organizations have warned that these devices generate aerosols containing nicotine and other potentially toxic substances [8]. Furthermore, available evidence suggests that they could affect biological pathways relevant to cardiometabolic pathophysiology [9]. Sex differences are especially relevant in the study of MetS [10], given that both the prevalence of its components and the patterns of exposure to conventional and electronic cigarettes can vary between men and women [11]. These differences could reflect not only differentiated consumption patterns, but also different forms of cardiometabolic vulnerability [12], making it pertinent to examine the association between these exposures and MetS from a sex-stratified perspective.

Despite growing concern regarding the cardiometabolic effects of nicotine exposure, evidence linking combustible cigarette smoking and electronic cigarette use to metabolic syndrome remains limited, particularly in working populations. This is relevant because occupational groups may exhibit specific patterns of exposure and cardiometabolic risk. In this context, the present study aimed to evaluate the association between combustible cigarette smoking, electronic cigarette use, and metabolic syndrome in a working population.

## 2. Materials and Methods

Sample selection and sample size

The study was conducted using an observational, descriptive, cross-sectional design among workers in the city of Popayan, Cauca, Colombia. Data collection was carried out in collaboration with 20 formal companies located in the urban area and representing different economic sectors, which authorized access to their employees during the fieldwork period. The eligible population consisted of workers with an active employment relationship in the participating companies, who voluntarily agreed to participate by signing written informed consent.

Inclusion and exclusion criteria

Participants were eligible for inclusion if they met the following criteria:

- Being an active worker in one of the participating formal companies located in the urban area of Popayán, Cauca, Colombia.
- Age of 18 years or older.
- Availability and presence during the data collection period.
- Voluntarily agreeing to participate in the study.
- Providing written informed consent prior to the administration of questionnaires and clinical or anthropometric assessments.

Participants were excluded if they met any of the following criteria:

- Absence of an active employment relationship with the participating company at the time of data collection.
- Refusal or inability to provide written informed consent.
- Incomplete information on tobacco exposure, electronic cigarette use, or the metabolic syndrome components required for classification according to International Diabetes Federation criteria.
- Missing information on variables required to classify tobacco exposure, electronic cigarette use, or metabolic syndrome.
- Pregnancy or any temporary clinical condition that could substantially alter anthropometric or metabolic measurements at the time of assessment.

These criteria were applied before the final analytical sample was defined, ensuring that all included participants had sufficient and valid information for exposure classification, metabolic syndrome diagnosis, and regression analyses.

Sample size was determined to ensure a 95% confidence level and a maximum margin of error of 4.71%. An expected proportion of 0.5 was assumed to provide a conservative estimate and to account for maximum variability in the target population.

Additionally, a 10% adjustment was incorporated to account for potential losses and incomplete records, in order to preserve the statistical precision of the estimates. Under these assumptions, the final required sample size was 822 participants, which was considered adequate to meet the objectives of the study.

Data collection procedures

After eligibility was confirmed according to the predefined inclusion and exclusion criteria, all participants provided written informed consent, authorizing the use of their data for research purposes under strict guarantees of confidentiality and anonymity. Subsequently, participants completed a structured questionnaire designed to collect sociodemographic and clinical variables relevant to the study.

Ethics statement

The study was conducted in accordance with the principles of the Declaration of Helsinki and Colombian regulations for health research involving human participants (Resolution 8430 of 1993, Ministry of Health of Colombia). The study protocol was approved by the Research Ethics Committee of the University of Cauca (Approval ID 5694, 29 May 2023). All participants were informed about the objectives of the study, the voluntary nature of their participation, the procedures involved, and the confidential use of the information collected. Written informed consent was obtained from all participants prior to enrollment. Participant confidentiality and anonymity were maintained throughout the study, and data were analyzed and reported in aggregate form.

Anthropometric assessments were conducted following the standardized protocols of the International Society for the Advancement of Kinanthropometry (ISAK) [13]. Body weight (kg) and height (cm) were measured using calibrated instruments, and body mass index (BMI) was calculated as weight divided by height squared (kg/m^2^). Nutritional status was classified according to conventional BMI thresholds as normal weight (<25 kg/m^2^), overweight (25–29.9 kg/m^2^), and obesity (≥30 kg/m^2^).

Definition of metabolic syndrome

Metabolic syndrome (MetS) was defined according to the criteria of the International Diabetes Federation (IDF) [14]. Waist circumference was measured using standardized procedures, and central obesity was defined as a waist circumference ≥90 cm in men and ≥80 cm in women, in accordance with the sex-specific thresholds recommended for the study population. Participants were classified as having MetS when central obesity was present together with at least two of the following abnormalities: systolic blood pressure ≥130 mmHg or diastolic blood pressure ≥85 mmHg, or previously diagnosed hypertension; triglycerides ≥150 mg/dL; HDL cholesterol <40 mg/dL in men or <50 mg/dL in women; or fasting plasma glucose ≥100 mg/dL, or previously diagnosed diabetes [15]. Blood pressure was measured using standardized techniques in accordance with the recommendations of the European Society of Hypertension [16]. For the present analysis, MetS status was recalculated from the individual components recorded in the database to ensure consistent application of the IDF criteria, and a binary outcome variable (MetS: yes/no) was created. In addition to the composite MetS outcome, each component was also examined separately as a secondary binary outcome, including central obesity, elevated blood pressure, hypertriglyceridemia, low HDL cholesterol, and impaired fasting glucose or diabetes.

Assessment of tobacco and electronic cigarette use

Tobacco exposure was assessed by interviewer-administered self-report using a structured questionnaire applied by trained study personnel during the data collection procedures. The instrument included items on conventional combustible cigarette smoking and electronic cigarette use. Current electronic cigarette use was defined as self-reported use on at least one day during the preceding 30 days. Based on this criterion, participants were classified as current users or non-current users. Combustible cigarette smoking was classified according to self-reported smoking history as never smoker versus current/former smoker. A third variable, dual use, was generated to identify participants who reported concurrent use of both electronic cigarettes and combustible cigarettes.

All smoking-related variables were coded categorically for analysis. The classification criteria used in the present study are the same as those reported in Table 1, but are presented here in the Methods section to ensure transparency and reproducibility of the exposure assessment.

Statistical analysis

All statistical analyses were performed using SPSS for Windows, version 26 (SPSS Inc., Chicago, IL, USA). Continuous variables were summarized as means and standard deviations (SD), and differences between study groups were assessed using Student's *t*-test. Categorical variables were described as frequencies and proportions, and differences in their distribution were evaluated using the chi-square test.

To examine the association between explanatory variables and metabolic syndrome (MetS), unconditional binary logistic regression was fitted to estimate crude odds ratios (ORs) and their corresponding 95% confidence intervals (95% CIs). To account for potential confounding, ORs were initially adjusted in a model including age (years, continuous variable) and sex (male vs. female) as covariates. In subsequent analyses, fully adjusted multivariable models were constructed incorporating additional categorical covariates, including occupational status, educational level, cigarette smoking, obesity, hypertension, diabetes, and dyslipidemia. Potential interactions between risk factors were further evaluated among variables that showed a significant association with the risk of MetS. Interaction analyses were conducted using SPSS macros implemented within the statistical software. A two-sided *p*-value < 0.05 was considered indicative of statistical significance for all analyses.

Exploratory interaction analyses

Additional interaction analyses were conducted on the multiplicative scale to explore whether the association between tobacco exposure variables and MetS varied according to the presence of individual metabolic components. These interaction analyses were not pre-specified in the original study protocol and should therefore be considered exploratory and hypothesis-generating. Given the number of interaction terms evaluated, *p*-values were interpreted with caution and adjusted for multiple comparisons using the Benjamini–Hochberg false discovery rate (FDR) procedure. Both nominal and FDR-adjusted results were examined, and emphasis was placed on the magnitude, precision, and biological plausibility of the observed associations rather than on statistical significance alone.

## 3. Results

### General Characteristics of the Study Sample

The study population comprised 822 workers employed across the participating organizations, which represented diverse sectors of economic activity and enabled the inclusion of a workforce with heterogeneous occupational contexts. The highest proportion of organizations belonged to the education sector (40%), followed by manufacturing industries (21%), transportation and storage (20%), and public administration and defense (19%). This sectoral distribution reflects the participation of organizations with distinct productive, service-related, and administrative profiles, supporting a more comprehensive epidemiological characterization of the study population. According to the diagnostic criteria established by the IDF, the prevalence of MetS in the study sample was 39.78% (327/822). Globally, MetS is estimated to affect approximately one quarter of the adult population and is substantially more common than diabetes mellitus.

This study also characterized the sociodemographic profile of workers with metabolic syndrome (Table 1). Among workers with MetS, men accounted for 63.0% of cases (206/327), whereas women accounted for 37.0%. In the comparison between workers with and without MetS, men were more frequently represented in the MetS group than in the non-MetS group (63.0% vs. 51.9%), and this difference was statistically significant (*p* = 0.002).

The median age of workers with MetS was 47 years and was similar between men and women. Age distribution did not differ significantly between workers with and without MetS (*p* = 0.192). However, workers aged 40 years or older represented a greater proportion of the MetS group than of the group without MetS (73.4% vs. 66.3%). In the age-stratified analysis, the highest frequency of MetS was observed among workers aged 40–49 years. Although this age group included a higher proportion of women, no statistically significant sex differences were observed across age groups (*p* = 0.466).

Educational attainment showed a non-significant trend across groups (*p* = 0.087). Lower levels of schooling, corresponding to primary or secondary education, were more frequent among workers with MetS than among those without the syndrome (39.8% vs. 30.7%). Monthly income was also evaluated as a sociodemographic characteristic. The distribution of income categories was different between workers with and without MetS, and the association was statistically significant (*p* = 0.002).

Neither exclusive e-cigarette use nor exclusive combustible cigarette use differed significantly between groups. Current e-cigarette use was reported in 16.5% of workers with MetS and in 14.5% of those without MetS (*p* = 0.443). Likewise, current or former combustible cigarette use was recorded in 26.6% of workers with MetS and in 21.6% of those without the syndrome (*p* = 0.145).

In contrast, dual use of e-cigarettes and combustible cigarettes was more common among workers without MetS than among those with the syndrome (18.4% vs. 7.0%, *p* < 0.001). Given the cross-sectional nature of the study, this finding should not be interpreted as evidence of a protective effect. Rather, it may reflect differences in smoking behavior after diagnosis, cessation patterns, or other unmeasured factors.

All four additional components of metabolic syndrome differed significantly between groups (all *p* < 0.001). Elevated blood pressure (≥130/85 mmHg) was present in 34.9% of workers with MetS, compared with 6.5% of those without the syndrome. Impaired fasting glucose (≥100 mg/dL) was observed in 17.7% of the MetS group and in 3.2% of the non-MetS group. Low HDL cholesterol was highly prevalent overall and was more frequent among workers with MetS than among those without MetS (84.1% vs. 48.7%). Hypertriglyceridemia (≥150 mg/dL) showed the largest between-group difference, affecting 79.8% of workers with MetS and 16.2% of those without the syndrome.

**Figure 1:**
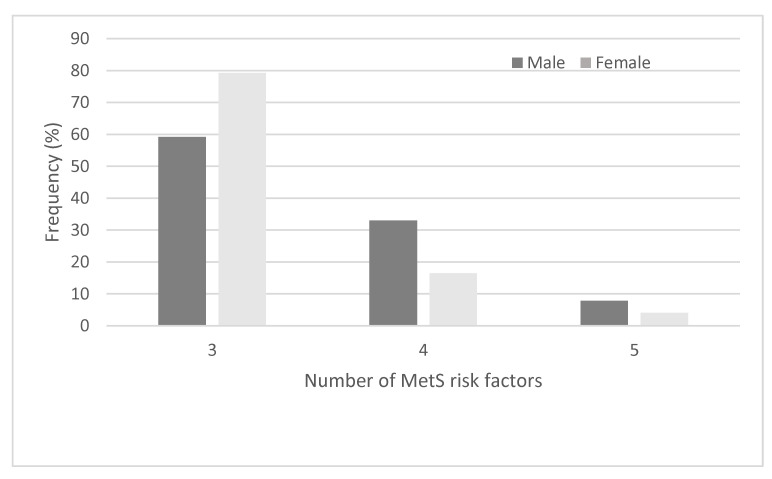
Distribution of number MetS risk factors in the study population.

Among the 327 workers diagnosed with MetS, all had increased waist circumference, as required by the IDF definition. Therefore, variation in metabolic burden within this group was determined by the accumulation of additional diagnostic criteria beyond central obesity. The distribution of this burden differed by sex (Figure 1). In both men and women, the largest proportion of cases met the minimum diagnostic threshold of three criteria. This pattern was more frequent among women than among men (79.3% vs. 59.2%).

By contrast, the proportion of workers meeting four criteria was higher in men than in women (33.0% vs. 16.5%). A similar pattern was observed for the most severe category, defined as the simultaneous presence of all five diagnostic criteria, which was identified in 7.8% of men and 4.1% of women. Overall, these findings suggest that male workers with MetS more frequently exhibited a greater accumulation of metabolic abnormalities than female workers.

Distribution of the number of MetS risk factors by sex among workers with metabolic syndrome (n = 327; 206 men, 121 women). All participants MetS the mandatory IDF criterion of increased waist circumference; values on the x-axis reflect abdominal obesity plus the indicated number of additional metabolic criteria. Percentages are calculated within each sex group.

**Figure 2:**
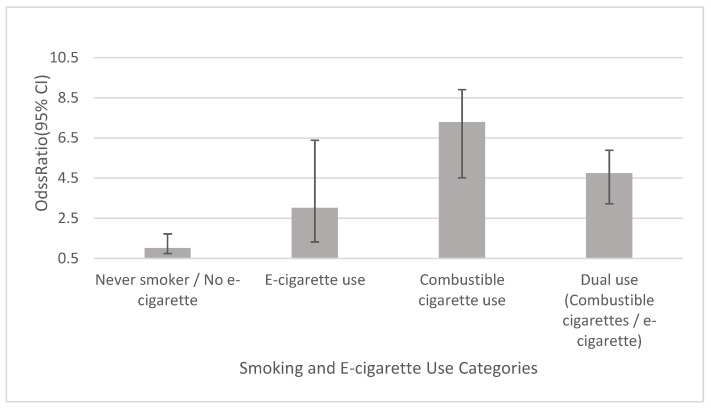
Odds ratios (ORs) and 95% confidence intervals according to categories of combustible cigarette smoking and e-cigarette use among patients with metabolic syndrome. The reference category consisted of participants who had never smoked combustible cigarettes and had never used e-cigarettes. Exclusive e-cigarette use showed an OR of 3.02, exclusive combustible cigarette use an OR of 7.29, and dual use an OR of 4.75, indicating a greater magnitude of association in categories with combustible cigarette exposure.

Consistent differences were observed in the magnitude of the association across categories of tobacco and e-cigarette use among patients with metabolic syndrome. Compared with participants who had never smoked combustible cigarettes and had never used e-cigarettes, the odds were higher in all exposed groups. Exclusive e-cigarette use showed an intermediate association (OR ≈ 3.02), whereas exclusive combustible cigarette use showed the greatest magnitude of association (OR ≈ 7.29). Dual users of combustible cigarettes and e-cigarettes also showed elevated odds (OR ≈ 4.75). Overall, these findings suggest a gradient of increasing risk across categories with combustible cigarette exposure, particularly among exclusive conventional cigarette smokers. Figure 2.

Exploratory interaction analyses on the multiplicative scale were performed to assess whether the association between tobacco exposure and metabolic syndrome (MetS) varied according to the co-occurrence of individual metabolic abnormalities (Table 2). Because a large number of interaction terms were evaluated, these analyses were considered hypothesis-generating and interpreted with appropriate caution. Among e-cigarette users, the strongest nominal interaction was observed with elevated blood pressure (OR = 9.4; 95% CI: 1.23–17.26; *p* = 0.031), and a similarly elevated estimate was found for the combination with elevated blood pressure and hypertriglyceridemia (OR = 8.1; 95% CI: 1.14–16.58; *p* = 0.035). Additional nominally significant pairwise interactions were identified with hyperglycemia (OR = 1.6; 95% CI: 1.14–2.37; *p* = 0.009), hypertriglyceridemia (OR = 2.6; 95% CI: 1.24–5.41; *p* = 0.011), and low HDL cholesterol (OR = 2.0; 95% CI: 1.12–4.11; *p* = 0.042), whereas the five-way interaction did not reach statistical significance (OR = 2.5; 95% CI: 0.88–6.96; *p* = 0.086). For combustible cigarette use, nominally significant interactions were observed across all pairwise combinations with MetS components, including elevated blood pressure (OR = 2.5; 95% CI: 1.38–4.52; *p* = 0.003), hyperglycemia (OR = 1.4; 95% CI: 1.20–1.82; *p* < 0.001), hypertriglyceridemia (OR = 2.0; 95% CI: 1.35–3.01; *p* < 0.001), and low HDL cholesterol (OR = 2.13; 95% CI: 1.42–3.21; *p* < 0.001). This pattern extended to higher-order terms, including the interaction with elevated blood pressure and low HDL cholesterol (OR = 3.03; 95% CI: 1.48–6.24; *p* = 0.003) and the five-component interaction term (OR = 2.12; 95% CI: 1.24–3.62; *p* = 0.006). Among dual users, the largest point estimates were concentrated in models involving elevated blood pressure, including the pairwise interaction term (OR = 11.5; 95% CI: 1.5–27.19; *p* = 0.018), the interaction with hypertriglyceridemia (OR = 9.4; 95% CI: 1.23–17.26; *p* = 0.031), and the interaction with low HDL cholesterol (OR = 10.8; 95% CI: 1.41–22.16; *p* = 0.022). By contrast, pairwise interaction terms for dual use with hyperglycemia, hypertriglyceridemia, and low HDL cholesterol in the absence of elevated blood pressure were not statistically significant (all *p* > 0.05). The four-way and five-way interaction terms for dual users were nominally significant (OR = 3.2; *p* = 0.037 and OR = 3.02; 95% CI: 1.04–8.84; *p* = 0.042, respectively). Overall, combustible cigarette use showed the most consistent interaction pattern across models, whereas dual use yielded the largest point estimates. However, the magnitude, precision, and stability of these estimates were heterogeneous, and several confidence intervals were wide. After adjustment for multiple comparisons using the Benjamini–Hochberg false discovery rate procedure, only the most robust interaction terms remained statistically noteworthy, whereas the remaining associations should be interpreted as exploratory signals requiring confirmation in independent studies.

## 4. Discussion

In this study conducted in southwestern Colombia, the observed prevalence of metabolic syndrome (MetS) was 40%, indicating a substantial cardiometabolic burden in this working population. This estimate is consistent with previous reports showing a high prevalence of MetS in occupational settings. Studies from Brazil and other workforce-based cohorts have documented similarly elevated frequencies, suggesting that certain occupational contexts may concentrate substantial cardiometabolic vulnerability [17], whereas Johnson et al. reported a prevalence of 44% among men in a university-based occupational cohort [18]. Likewise, a recent study among teachers in South Africa found a prevalence of 58%, further supporting the notion that specific occupational environments may concentrate a substantial cardiometabolic burden [19]. From a practical perspective, four out of every ten participants met the diagnostic criteria for MetS, underscoring the magnitude of this problem in an economically active population.

MetS prevalence was higher in men, in agreement with previous studies in occupational populations that have documented a greater frequency of this cardiometabolic phenotype among male workers [20]. An additional finding of this study was that men more frequently accumulated four to five MetS components, whereas women more often clustered at the minimum diagnostic threshold of three components according to the International Diabetes Federation criteria. This pattern suggests that sex-related differences may exist not only in the occurrence of the syndrome but also in its clinical and metabolic expression. These findings are consistent with previous reports describing a greater accumulation of metabolic abnormalities among men [21,22,23].

With respect to age, metabolic syndrome was more frequent among workers older than 40 years. This finding is consistent with existing evidence showing that the probability of developing MetS increases progressively with age. In this context, aging may favor the accumulation of metabolic abnormalities through the interaction of physiological changes, long-term lifestyle patterns, and prolonged exposure to cardiometabolic risk factors. Overall, these results support the interpretation that, in working populations, sex and age are important axes in the distribution of cardiometabolic risk. The higher frequency of MetS in men and older workers underscores the need to strengthen screening, surveillance, and targeted prevention strategies within occupational settings [3,10,24].

Regarding tobacco exposure, the findings indicate that its contribution to MetS may extend beyond that of an isolated behavioral risk factor [20]. Combustible cigarette smoking exhibited the most consistent pattern across models, whereas dual use showed the largest effect estimates in selected analyses, particularly those involving elevated blood pressure [25]. The lower descriptive frequency of dual use among workers with MetS should not be interpreted as evidence of a protective or inverse association, as the cross-sectional design precludes establishing temporality or causality [26]. Even so, the observed pattern suggests that dual use may remain a clinically relevant exposure in the configuration of cardiometabolic risk.

Within this framework, it is particularly noteworthy that electronic cigarette use also showed significant interactions with specific metabolic components, suggesting that its cardiometabolic effects may not be negligible in working populations [27]. Taken together, these findings indicate that tobacco exposure, whether combustible, electronic, or dual, may act as a modulator of cardiometabolic clustering by amplifying the interdependence between dyslipidemia, glycemic alterations, and hemodynamic burden [28]. The prominent role of elevated blood pressure as a possible effect modifier, especially among dual users and electronic cigarette users, reinforces the hypothesis that nicotine and other tobacco-related compounds may potentiate vascular and metabolic damage when an underlying pathophysiological susceptibility is already present [29]. From this perspective, tobacco use may not operate in isolation, but rather as a factor that intensifies the internal architecture of cardiometabolic risk [27].

In addition, the metabolic profile of this population was dominated by an atherogenic dyslipidemic pattern, characterized by a high frequency of hypertriglyceridemia and low HDL cholesterol, which were the components showing the greatest differences between workers with and without MetS. This finding is epidemiologically relevant because it suggests that, in this occupational population, MetS is not expressed solely as a clustering of abdominal adiposity and glucohemodynamic alterations, but rather as a systemic disorder in which dyslipidemia plays a central role. The high frequency of low HDL cholesterol even outside the MetS group, together with the marked contrast in elevated triglycerides, supports the hypothesis of an underlying metabolic background characterized by lipid dysfunction, possible insulin resistance, and low-grade inflammation, which may be further aggravated by concurrent behavioral and occupational exposures. From a public health perspective, this pattern highlights the need for workplace-based preventive strategies focused not only on overall MetS screening, but also on the early identification and systematic follow-up of lipid abnormalities as early signals of cardiometabolic deterioration [30,31,32].

Overall, the findings of this study support an integrated interpretation of metabolic syndrome in this working population from southwestern Colombia as a clinical phenotype characterized predominantly by atherogenic dyslipidemia, greater cumulative severity among men, and a potentially unfavorable cardiometabolic profile associated with tobacco-related exposures, particularly in the context of dual use and elevated blood pressure [33].

The present study has several limitations that should be considered when interpreting the results. First, its cross-sectional design precludes the establishment of temporal or causal relationships between exposure to combustible cigarette smoking, electronic cigarette use, and the presence of metabolic syndrome. Therefore, the observed associations should be interpreted as epidemiological relationships rather than evidence of causality. This limitation is particularly relevant in the analysis of dual use, as consumption patterns may have changed after the diagnosis or recognition of metabolic abnormalities. Second, exposure to tobacco and electronic cigarettes was assessed through self-report, which may introduce information bias, particularly due to underreporting, recall bias, or misclassification of participants.

Third, although the sample included 822 workers from 20 formal organizations in Popayán, Cauca, participant selection was conditioned by institutional acceptance and voluntary worker participation. Consequently, the findings may not be fully generalizable to informal workers, unemployed individuals, rural populations, or other occupational settings with different labor, social, and environmental conditions. Finally, interaction analyses should be interpreted with caution, given their exploratory nature and the large number of terms evaluated.

## 5. Conclusions

MetS was highly prevalent in this working population from southwestern Colombia, affecting nearly four out of ten participants and revealing a substantial cardiometabolic burden in an economically active group. Its expression was characterized predominantly by an atherogenic dyslipidemic pattern, particularly hypertriglyceridemia and low HDL cholesterol, while men showed a greater accumulation of metabolic abnormalities than women. Taken together, these findings indicate that, in this occupational setting, MetS is not merely a clustering of isolated risk factors, but rather a clinically relevant and heterogeneous cardiometabolic phenotype with marked implications for long-term cardiovascular health.

In addition, tobacco-related exposures were consistently associated with a less favorable cardiometabolic profile. Although combustible cigarette smoking showed the most robust overall pattern of association with MetS, both electronic cigarette use and dual use also exhibited unfavorable effect estimates, supporting the view that these exposures may contribute to the clustering of metabolic abnormalities rather than acting as independent behavioral correlates alone. The particularly elevated estimates observed in analyses involving blood pressure further suggest that tobacco exposure may interact with existing metabolic vulnerability and intensify cardiometabolic risk in susceptible workers. Given the cross-sectional design, these findings should be interpreted cautiously and confirmed in longitudinal studies.

From an epidemiological and occupational health perspective, these findings suggest that the workplace should not be viewed solely as a setting for conventional preventive interventions, but also as a critical environment for the early identification of particularly unfavorable cardiometabolic risk profiles. The coexistence of an atherogenic dyslipidemic pattern with tobacco-related exposures indicates that some workers may have a more concentrated and complex metabolic vulnerability than that captured by routine screening strategies. In this context, occupational health programs may benefit from more selective approaches aimed at the early recognition of higher-susceptibility profiles and timely intervention before progression to cardiovascular disease or type 2 diabetes.

## Figures and Tables

**Table 1:** Sociodemographic and clinical characteristics by sex in workers with MetS.

Characteristics		Without MetS n = 495	With MetS n = 327	*p*
Age	18–39	167 (33.7)	87 (26.6)	0.192
	40–49	176 (35.6)	126 (38.5)	
	>50	152 (30.7)	114 (34.9)	
Sex	Male	257 (51.9)	206 (63.0)	0.002
	Female	238 (48.1)	121 (37.0)	
Educational level	Primary/Secondary Education	152 (30.7)	130 (39.8)	0.087
	Technical/Technological	117 (23.6)	69 (21.1)	
	University	226 (45.7)	128 (39.1)	
Type of employment	Public	265 (53.5)	185 (56.6)	0.391
	Private	230 (46.5)	142 (43.4)	
Monthly household income level	Low income (<1 minimum wage)	50 (10.1)	60 (18.3)	0.002
	Middle income (1–3 minimum wages)	321 (64.8)	201 (61.5)	
	High income (>3 minimum wages)	124 (25.1)	66 (20.2)	
Physical activity	No	228 (46.1)	145 (44.3)	0.628
	Yes	267 (53.9)	182 (55.7)	
Electronic cigarette use	Never	423 (85.5)	273 (83.5)	0.443
	Current	72 (14.5)	54 (16.5)	
Combustible Cigarettes	Never	388 (78.4)	240 (73.4)	0.145
	Current/former	107 (21.6)	87 (26.6)	
Dual users (e-cigarettes + combustible cigarettes)	Never	404 (81.6)	304 (93.0)	<0.001
	Current	91 (18.4)	23 (7.0)	
Blood pressure	Normal (<130/85 mmHg)	463 (93.5)	213 (65.1)	<0.001
	Elevated (≥130/85 mmHg)	32 (6.5)	114 (34.9)	
Glucose level	Normal (<100 mg/dL)	479 (96.8)	269 (82.3)	<0.001
	Elevated (≥100 mg/dL)	16 (3.2)	58 (17.7)	
HDL cholesterol concentration	Normal (>40 men; >50 women)	254 (51.3)	52 (15.9)	<0.001
	Low (<40 men; <50 women)	241 (48.7)	275 (84.1)	
Triglyceride concentration	Normal (<150 mg/dL)	415 (83.8)	66 (20.2)	<0.001
	Elevated (≥150 mg/dL)	80 (16.2)	261 (79.8)	

Data are presented as n (%). Chi-square test was used for group comparisons. Statistical significance was set at *p* < 0.05 (two-tailed). “Current” tobacco use was defined as use on at least one day in the preceding 30 days; dual users reported concurrent use of both e-cigarettes and combustible cigarettes. MetS was defined per IDF criteria. Abbreviations: HDL, high-density lipoprotein; IDF, International Diabetes Federation; MetS, metabolic syndrome. Income was categorized based on multiples of the Colombian national monthly minimum wage (SMLV) at the time of data collection. Low income: <1 SMLV; middle income: 1–3 SMLV; high income: >3 SMLV. Abbreviations: MetS, metabolic syndrome; IDF, International Diabetes Federation.

**Table 2:** Exploratory multiplicative interaction analyses between tobacco exposure categories and selected metabolic components in relation to metabolic syndrome.

Study Variable	Crude OR (95% CI)	*p*	Adjusted OR (95% CI)	*p*
Electronic cigarette use*Elevated blood pressure	9.4 (1.23–17.26)	0.031	9.5 (1.24–17.11)	0.034
Electronic cigarette use*hyperglycemia	1.6 (1.14–2.37)	0.009	1.6 (1.13–2.36)	0.009
Electronic cigarette use*Hypertriglyceridemia	2.6 (1.24–5.41)	0.011	2.6 (1.22–5.35)	0.012
Electronic cigarette use*Low c-HDL	2.0 (1.12–4.11)	0.042	2.0 (1.16–4.01)	0.045
Electronic cigarette use*Elevated blood pressure* hyperglycemia	3.5 (1.23–9.84)	0.019	3.4 (1.21–9.67)	0.021
Electronic cigarette use*Elevated blood pressure* Hypertriglyceridemia	8.1 (1.14–16.58)	0.035	8.1 (1.14–16.58)	0.036
Electronic cigarette use*Elevated blood pressure* Low c-HDL	7.42 (0.95–57.81)	0.056	7.5 (0.95–58.22)	0.055
Electronic cigarette use*hyperglycemia*Hypertriglyceridemia	1.6 (1.13–2.41)	0.009	1.6 (1.12–2.40)	0.010
Electronic cigarette use*hyperglycemia*Low c-HDL	1.5 (1.16–2.23)	0.022	1.5 (1.15–2.22)	0.025
Electronic cigarette use*Hypertriglyceridemia* Low c-HDL	2.27 (1.18–4.77)	0.030	2.24 (1.16–4.72)	0.033
Electronic cigarette use*Elevated blood pressure* hyperglycemia*Hypertriglyceridemia	2.8 (1.12–7.91)	0.033	2.8 (1.12–7.94)	0.034
Electronic cigarette use*Elevated blood pressure* hyperglycemia*Hypertriglyceridemia*Low c-HDL	2.5 (0.88–6.96)	0.086	2.48 (0.88–6.98)	0.085
Combustible Cigarettes*Elevated blood pressure	2.5 (1.38–4.52)	0.003	2.5 (1.39–4.58)	0.002
Combustible Cigarettes*hyperglycemia	1.4 (1.20–1.82)	<0.001	1.5 (1.21–1.83)	<0.001
Combustible Cigarettes*Hypertriglyceridemia	2.0 (1.35–3.01)	<0.001	2.0 (1.34–3.03)	<0.001
Combustible Cigarettes*Low c-HDL	2.13 (1.42–3.21)	<0.001	2.15 (1.43–3.24)	<0.001
Combustible Cigarettes*Elevated blood pressure* hyperglycemia	1.6 (1.16–2.22)	0.004	1.6 (1.17–2.23)	0.004
Combustible Cigarettes *Elevated blood pressure* Hypertriglyceridemia	2.7 (1.33–5.69)	0.006	2.8 (1.34–5.75)	0.006
Combustible Cigarettes *Elevated blood pressure* Low c-HDL	3.03 (1.48–6.24)	0.003	3.01 (1.49–6.33)	0.002
Combustible Cigarettes * hyperglycemia * Hypertriglyceridemia	1.2 (1.05–1.31)	0.005	1.2 (1.05–1.32)	0.004
Combustible Cigarettes * Elevated blood pressure* hyperglycemia* Hypertriglyceridemia	1.7 (1.16–2.56)	0.007	1.7 (1.16–2.57)	0.007
Combustible Cigarettes * Elevated blood pressure* hyperglycemia* Hypertriglyceridemia* Low c-HDL	2.12 (1.24–3.62)	0.006	2.13 (1.24–3.64)	0.006
Dual users * (e-cigarettes + combustible cigarettes)* Elevated blood pressure	11.5 (1.5–27.19)	0.018	11.6 (1.5–28.04)	0.018
Dual users (e-cigarettes + combustible cigarettes)* hyperglycemia	1.7 (0.92–2.97)	0.090	1.7 (0.92–2.98)	0.089
Dual users (e-cigarettes + combustible cigarettes)* hypertriglyceridemia	2.3 (0.74–7.09)	0.148	2.3 (0.74–7.13)	0.147
Dual users (e-cigarettes + combustible cigarettes)* Low c-HDL	2.1 (0.75–5.81)	0.16	2.0 (0.75–5.85)	0.158
Dual users (e-cigarettes + combustible cigarettes)* Elevated blood pressure* Hyperglycemia	3.5 (1.20–10.46)	0.022	3.5 (1.20–10.49)	0.022
Dual users (e-cigarettes + combustible cigarettes)* Elevated blood pressure* hypertriglyceridemia	9.4 (1.23–17.26)	0.031	9.5 (1.24–17.79)	0.031
Dual users (e-cigarettes + combustible cigarettes)* Elevated blood pressure* Low c-HDL	10.8 (1.41–22.16)	0.022	10.9 (1.43–22.85)	0.021
Dual users (e-cigarettes + combustible cigarettes)* hyperglycemia * Hypertriglyceridemia	1.6 (0.85–2.96)	0.149	1.6 (0.85–2.96)	0.147
Dual users (e-cigarettes + combustible cigarettes)* Elevated blood pressure* hyperglycemia* Hypertriglyceridemia	3.2 (1.07–9.53)	0.038	3.2 (1.07–9.54)	0.037
Dual users (e-cigarettes + combustible cigarettes)* Elevated blood pressure* hyperglycemia* Hypertriglyceridemia* Low c-HDL	3.02 (1.04–8.84)	0.043	3.03 (1.04–8.85)	0.042

CI: confidence interval; MetS: metabolic syndrome; OR: odds ratio. Adjusted for age and Educational level; *: denotes the multiplicative interaction between the indicated variables.

## Data Availability

The original contributions presented in this study are fully included within the article itself. All key findings, analyses, and materials necessary to understand the scope and outcomes of the research are provided in the main text and/or its supplementary sections. Therefore, readers can refer directly to the article for the essential information related to the study. Any further inquiries, requests for clarification, or additional information may be directed to the corresponding author, who can provide further details when appropriate.

## Abbreviations

The following abbreviations are used in this manuscript:

MetS	Metabolic Syndrome
CVD	Cardiovascular disease
HDL	High-density lipoprotein
T2DM	Type 2 diabetes mellitus
ISAK	International Society for the Advancement of Kinanthropometry.
BMI	Body mass index
IDF	International Diabetes Federation
